# Diversity of bacterial symbionts associated with *Myzus persicae* (Sulzer) (Hemiptera: Aphididae: Aphidinae) revealed by 16S rRNA Illumina sequencing

**DOI:** 10.1007/s00248-020-01622-6

**Published:** 2020-10-17

**Authors:** Shifen Xu, Liyun Jiang, Gexia Qiao, Jing Chen

**Affiliations:** 1 Key Laboratory of Zoological Systematics and Evolution, Institute of Zoology, Chinese Academy of Sciences, Beijing, 100101 China; 2 College of Life Sciences, University of Chinese Academy of Sciences, Beijing, 100049 China

**Keywords:** High-throughput 16S rRNA gene sequencing, Horizontal transmission, Polyphagous aphid, Symbiont community

## Abstract

Aphids are known to be associated with a variety of symbiotic bacteria. To improve our knowledge of the bacterial diversity of polyphagous aphids, in the present study, we investigated the microbiota of the cosmopolitan agricultural pest *Myzus persicae* (Sulzer). Ninety-two aphid samples collected from different host plants in various regions of China were examined using high-throughput amplicon sequencing. We comprehensively characterized the symbiont diversity of *M. persicae* and assessed the variations in aphid-associated symbiont communities. We detected a higher diversity of symbionts than has been previously observed. *M. persicae* hosted the primary endosymbiont *Buchnera aphidicola* and seven secondary symbionts, among which *Wolbachia* was the most prevalent and *Rickettsia*, *Arsenophonus*, and *Spiroplasma* were reported for the first time. Ordination analyses and statistical tests revealed that the symbiont flora associated with *M. persicae* did not change with respect to host plant or geography, which may be due to frequent migrations between different aphid populations. These findings will advance our knowledge of the microbiota of polyphagous insects and will enrich our understanding of assembly of host-microbiome systems.

**Electronic supplementary material:**

The online version of this article (10.1007/s00248-020-01622-6) contains supplementary material, which is available to authorized users.

## Introduction

Bacterial symbionts are widespread in insects, and their symbiotic associations range from obligate mutualism to facultative parasitism [1]. Xylem or phloem sap-feeding insect lineages typically host obligate symbionts that supply the nutrients necessary to supplement their unbalanced sap diets [2]. One typical example of obligate mutualism is the symbiosis between aphids and *Buchnera aphidicola*. *Buchnera* is the primary endosymbiont of aphids, resides in bacteriocytes, and can provide essential amino acids and vitamins for its hosts [3, 4]. Because of its strict vertical transmission, *Buchnera* has undergone long-term coevolution with its aphid hosts [5]. However, due to the metabolic losses caused by rapid genome deterioration in *Buchnera* [6], some aphid species of the subfamilies Lachninae, Chaitophorinae, and Aphidinae have established co-obligate associations with other symbiotic partners [7, 8].

In addition to the primary endosymbiont, aphids also carry a variety of secondary or facultative symbionts that are not required for aphid survival or reproduction but may confer fitness benefits. Secondary symbionts inhabit bacteriocytes, sheath cells, or hemocoel [9] and are maternally or horizontally transmitted [10]. Nine secondary symbionts have been widely reported in aphids, namely, *Serratia symbiotica*, *Rickettsia*, *Wolbachia*, *Hamiltonella defensa*, *Regiella insecticola*, *Spiroplasma*, *Arsenophonus*, *Fukatsuia symbiotica*, and *Rickettsiella viridis*. Secondary symbionts can provide various ecological benefits to aphids, including conferring resistance to parasitic wasps and fungal pathogens [11, 12], improving tolerance to thermal stress [13], manipulating aphid reproduction [14], affecting host plant utilization [15], and even altering aphid body color [16].

Individual aphid species generally exhibit symbiont variation at the population level. Many studies using diagnostic PCR have observed differences in symbiont composition and prevalence with respect to host plant, geography, and season in a given aphid species [17–21]. However, such PCR-based detection techniques are limited in that they depend on prior knowledge of symbiont diversity. In addition, it is important to elucidate the determinants of symbiont community structure based on a large number of samples that represent different environmental factors. In recent years, high-throughput amplicon sequencing has become an effective method for understanding insect microbial communities and their dynamics. Although high-throughput sequencing techniques have been used by many studies to investigate the bacterial communities of specific aphid groups [22, 23] and community changes within some aphid species [24, 25], the symbiont diversity of most aphid species remains unknown or poorly characterized.

The green peach aphid, *Myzus persicae* (Sulzer), is a cosmopolitan agricultural pest. *M. persicae* is heteroecious and holocyclic, alternating between its primary hosts (plants of the genera *Amygdalus* and *Prunus*) and more than 40 families of secondary host plants [26]. In the tropics and regions where the primary host is absent, *M. persicae* can also live parthenogenetically on secondary host plants all the year round. In addition, *M. persicae* is an important vector of many plant viruses. However, to date, few studies have investigated the bacterial diversity of *M. persicae*. In a survey study by Henry et al. [27], only three of 50 individual *M. persicae* were shown to be infected with *H. defensa*, *R. insecticola*, or *S. symbiotica*. Chen et al. [28] observed a high prevalence of *Wolbachia* in *M. persicae* from China and revealed geographic variation in its prevalence and infection pattern. Using high-throughput sequencing, Gallo-Franco et al. [29] examined the bacterial communities of *M. persicae* feeding on the pepper crop *Capsicum annuum* from two localities of southwestern Colombia and identified that *Buchnera* was the predominant symbiont, but they did not report any secondary symbionts.

To better understand the microbial profiles of *M. persicae* and to verify whether this species harbors a low diversity of symbionts as revealed by previous studies, using 16S rRNA amplicon Illumina sequencing, we characterized the bacterial diversity of *M. persicae* with a broad sampling from different host plants throughout China. The bacterial community dynamics over aphid populations was also evaluated.

## Materials and Methods

### Sampling and DNA Isolation

**Fig. 1:**
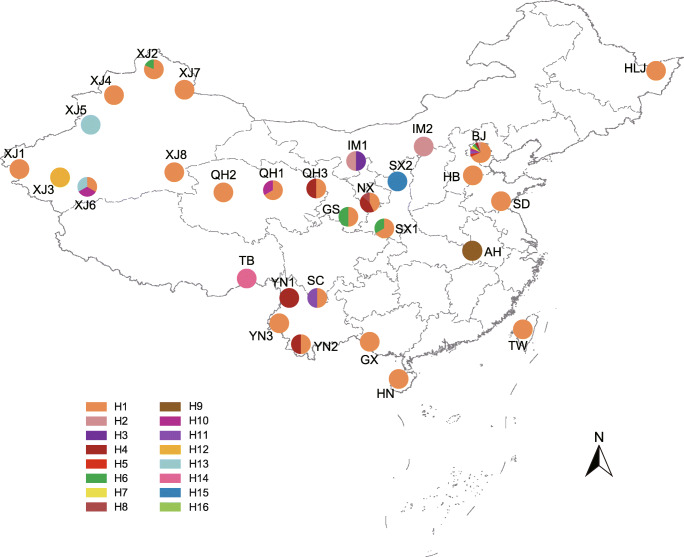
Collection regions and haplotype distribution of *Myzus persicae*. The haplotypes (H1–H16) are colored and labeled on the map. The abbreviations of geographic regions are provided in Table S2

Ninety-two samples of *M. persicae* were collected from 16 families of plants in 30 geographic regions of China between 2002 and 2016 (Fig. 1, Table S1). Specimens preserved in 75% ethanol were used to make voucher specimens, and samples preserved in 100% ethanol at − 20 °C were used for DNA isolation. Species identification of the voucher slides was performed based on morphological characteristics. All samples were deposited in the National Zoological Museum of China, Institute of Zoology, Chinese Academy of Sciences, Beijing, China.

The whole body of an adult aphid from a sample was used to extract total DNA, before which the aphid was rinsed in 70% ethanol for 5 min and then rinsed four times with sterilized water to eliminate the external microbial contaminants. DNA was isolated using the DNeasy Blood & Tissue Kit (QIAGEN, Germany). A sample of sterilized water was also included in the extraction procedure as a negative extraction control. All DNA extracts were stored at − 20 °C. To verify the aphid species identification, test the DNA quality, and exclude parasitized samples, COI barcodes were amplified using the universal primers LCO1490 and HCO2198 [30].

### 16S rRNA Amplification and Sequencing

A nearly 420-bp V3–V4 region of 16S rRNA gene was amplified with the primers 338F (5′-ACTCCTACGGGAGGCAGCA-3′) and 806R (5′-GGACTACHVGGGTWTCTAAT-3′) [31]. Two polymerase chain reaction (PCR) procedures were performed, where the first reaction generated the primary 16S amplicons and the second converted the amplicons into libraries for sequencing. The first-step PCR mixture consisted of 10 μL 5× Q5 Reaction Buffer (New England Biolabs, USA), 0.4 μL Q5 High-Fidelity DNA Polymerase (New England Biolabs), 1 μL dNTPs, 10 μL 5× Q5 High GC Enhancer (New England Biolabs), 1.5 μL forward and reverse primers (10 μM), and 40–60 ng DNA. PCR amplification was performed using the following thermocycling program: a 5-min initial denaturation at 95 °C followed by 15 cycles of 1 min at 95 °C, 1 min at 50 °C, and 1 min at 72 °C, and a final elongation for 7 min at 72 °C. In the second-step PCR, the forward primer (5′-AATGATACGGCGACCACCGAGATCTACAC-NNNNNNNN-ACACTCTTTCCCTACACGACGCTCTTCCGATCT-3′) contained the Illumina i5 adapter, a unique 8-bp barcode sequence for each sample (indicated in N), and read 1 sequencing primer binding sites; the reverse primer (5′-CAAGCAGAAGACGGCATACGAGAT-NNNNNNNN-GTGACTGGAGTTCAGACGTGTGCTCTTCCGATCT-3′) included the Illumina i7 adapter, a unique 8-bp barcode sequence for each sample (indicated in N), and read 2 sequencing primer binding sites. The reaction mixture contained 10 μL purified product from the first PCR, 20 μL 2× Phusion High-Fidelity PCR Master Mix (New England Biolabs) and 1 μL forward and reverse primers (10 μM). The reaction was heated for 30 s at 98 °C and then underwent 10 cycles of denaturation for 10 s at 98 °C, annealing for 30 s at 65 °C, and elongation for 30 s at 72 °C, with a final extension for 5 min at 72 °C. Samples of sterilized water were included in all PCRs as negative amplification controls. The PCR products were checked with 1.8% agarose gel electrophoresis. The positive samples were purified and mixed at a mass ratio of 1:1, after which the library was sequenced on an Illumina HiSeq 2500 platform, and 250-bp paired-end reads were generated.

### Bioinformatics Analyses

Both operational taxonomic unit (OTU) picking and amplicon sequence variant (ASV) approaches were used in the present study. For OTU clustering, paired-end reads were first merged using FLASH v1.2.11 [32]. The raw reads with an average quality score below 20 in a 50-bp sliding window were trimmed using Trimmomatic v0.33 [33], and reads shorter than 300 bp were removed. Chimeras were filtered by UCHIME v8.1 [34]. The resulting high-quality clean reads were clustered using the QIIME UCLUST module [35], and reads with a similarity ≥ 97% were grouped into an OTU. ASVs were generated using the DADA2 pipeline [36]. The first eight nucleotides of each read were trimmed, and the forward and reverse reads were then truncated at 245 and 200 bp, respectively. We removed low-quality reads with default filtering parameters and merged paired-end reads. Chimeric sequences were filtered using the function *removeBimeraDenovo*. The OTU representative sequences and ASV sequences were aligned with the SILVA reference database v.128 for taxonomy annotation. Sequences identified as chloroplasts, mitochondria, or eukaryotes, and all OTUs/ASVs with an abundance less than 0.005% were filtered, unless they could be annotated [37]. The resulting OTU and ASV count matrices included 22,032–66,994 and 4919–32,368 sequences per sample, respectively. For both data sets, we rarefied all samples to the median number of sequences per sample prior to downstream analyses.

### Statistical Analyses

Based on the OTU data set, Shannon and Simpson indices were calculated with the function *diversity* of the *vegan* package [38] in R v3.4.3 [39] to characterize the alpha diversity. Both indices are commonly used in estimating microbial diversity, with the Shannon index giving greater weight to richness, while the Simpson index stressing evenness [40]. The relative abundances of individual bacterial taxa were estimated by total sum scaling normalization with the function *decostand* in *vegan*. To assess variations in the symbiont and secondary symbiont communities with respect to distinct factors, we grouped all *M. persicae* samples according to host-plant family and geographic region (Table S2). First, variations in alpha diversity were examined. Because the alpha diversity data were not normally distributed (Shapiro–Wilk test: *P* < 0.05), two nonparametric methods Kruskal–Wallis test and Wilcoxon tests were performed to test for differences across all groups and between pairwise groups (*n* ≥ 3), respectively.

Then, we used the Bray–Curtis distances of relative abundance and presence/absence data to quantify the variations in symbiont and secondary symbiont communities among samples and examined how beta-diversity patterns were related to the host plant and geography. The Bray–Curtis dissimilarities were calculated based on both OTU and ASV data sets with the function *vegdist* of *vegan*. We logarithmically transformed the relative abundance data of the symbiont community (function *decostand* in *vegan*) to balance the bias caused by the most abundant *Buchnera*.

We used ordination analyses and statistical tests to detect whether there were changes in the symbiont and secondary symbiont community structures. We conducted unconstrained nonmetric multidimensional scaling (NMDS; function *metaMDS* of *vegan*; stress values < 0.05 were considered indicative of excellent representations) and constrained principal coordinate analysis (cPCoA; functions *capscale* and *anova.cca* of *vegan*) to visualize the Bray–Curtis dissimilarity. These two ordination methods are useful for summarizing community changes in relation to environmental factors. We performed analysis of similarities (ANOSIM) and permutational multivariate analysis of variance (PERMANOVA) with the Bray–Curtis dissimilarity to assess differences in the symbiont and secondary symbiont community compositions. The statistical tests were implemented using the functions *anosim* and *adonis* in *vegan* with 999 permutations to test for significance. ANOSIM and PERMANOVA are both widely used in microbiome studies, with the latter typically being more robust [41]. We then examined the relative abundance of each symbiont defined by OTU picking using the nonparametric Kruskal–Wallis test to further identify which symbiont contributed to the community variation. Fisher’s least significant difference (LSD) post hoc tests with a Bonferroni correction were performed for pairwise group comparisons of the relative abundances of specific symbionts. These two tests were applied using the function *kruskal* in *agricolae* [42].

Finally, we used Mantel tests with the Pearson correlation coefficient (function *mantel* in *vegan*) to examine whether the symbiont and secondary symbiont community changes were associated with spatial distance. The Mantel test determined the amount of correlation between the geographic distance matrix and the Bray–Curtis dissimilarity of the OTU data through a permutation procedure. The distances among sampling sites were calculated with Geographic Distance Matrix Generator v1.2.3 [43].

## Results

### Bacterial Community Profiling

**Fig. 2:**
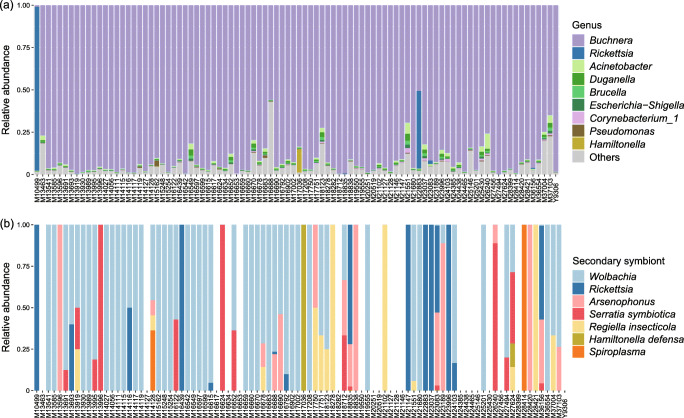
Bar plots of microbial communities associated with *Myzus persicae* across all samples. **a** Bacterial communities. **b** Secondary symbiont communities. Each bar shows the relative abundances of bacteria detected in each aphid sample

After filtering the noisy raw data, a total of 3,219,756 reads (34,997 reads per sample) were assigned to 1505 OTUs. The bacterial communities of *M. persicae* were dominated by the phylum Proteobacteria (average relative abundance across all samples: 96.83%), with other phyla accounting for less than 1% (Table S3). At the family level, the most abundant OTUs were associated with Enterobacteriaceae (91.37%). At the genus level, *Buchnera* was the most represented taxon (90.77%), followed by *Rickettsia* (1.59%), *Acinetobacter* (0.98%), and *Duganella* (0.56%) (Fig. 2, Table S3). The alpha diversity estimates of bacteria associated with all *M. persicae* samples ranged from 0.080 to 3.185 for the Shannon index and from 0.308 to 0.983 for the Simpson index (Table S4).

### Core Symbiont Community

Eight common symbionts of aphids were detected in *M. persicae*, with *B. aphidicola* detected in each sample at an extremely high relative abundance (90.77%) (Fig. 2a, Table 1). Secondary symbionts were detected within 72 out of 92 samples (Fig. 2b). Single OTUs were identified for *Rickettsia*, *S. symbiotica*, *R. insecticola*, *H. defensa*, and *Spiroplasma*. Two and three OTUs of *Wolbachia* and *Arsenophonus* were detected, respectively, where each OTU was shared between different aphid samples, and multiple OTUs of *Wolbachia* or *Arsenophonus* were detected in individual aphids for a few samples. *Wolbachia* was the most prevalent secondary symbiont (infection frequency: 53/92), followed by *Rickettsia* (15/92), *Arsenophonus* (15/92), *S. symbiotica* (15/92), and *R. insecticola* (12/92) (Table 1). *H. defensa* and *Spiroplasma* were only detected in four and three samples, respectively.

**Table 1:** The infection frequencies and average relative abundances of symbionts in *Myzus persicae*

Symbiont	Infection frequency	Relative abundance across all samples (%)	Relative abundance across infected samples (%)
*Buchnera aphidicola*	92/92	90.773	90.733
*Wolbachia*	53/92	0.015	0.027
*Rickettsia*	15/92 (13/92)	1.587 (0.036)	9.733 (0.251)
*Arsenophonus*	15/92	0.027	0.167
*Serratia symbiotica*	15/92	0.003	0.018
*Regiella insecticola*	12/92	0.001	0.005
*Hamiltonella defensa*	4/92	0.150	3.450
*Spiroplasma*	3/92	< 0.001	0.006

Values in parentheses were calculated without counting samples M10499 and M22883

The abundances of secondary symbionts were low relative to *Buchnera*, with average relative abundances across all samples far below 1%, except that of *Rickettsia* (1.59%) (Table 1). This result was attributed to the high abundance of *Rickettsia* in two aphid samples, M10499 (96.84%) and M22883 (45.89%), feeding on *Crepidiastrum sonchifolium* (Fig. 2a)*.* This finding was further supported by three biological replicates (i.e., three aphid individuals from one colony) and two PCR replicates (56.14–78.76%) (Fig. S1). The relative abundance of *Rickettsia* across all samples remained 0.04% when these two samples were not counted. The alpha diversity estimates of symbiont communities within all *M. persicae* samples ranged from 0.023 to 0.717 for the Shannon index and from 0.498 to 0.995 for the Simpson index. After excluding the primary endosymbiont *Buchnera*, the alpha diversity estimates of secondary symbiont communities ranged from 0 to 1.468 for the Shannon index and from 0 to 1 for the Simpson index (Table S4).

### Variations in Symbiont and Secondary Symbiont Communities

In general, the host plant and geography appeared to have no significant impacts on the symbiont flora of *M. persicae*. The Kruskal–Wallis and Wilcoxon tests based on the OTU data set revealed no variation in the alpha diversity of the symbiont community in relation to the host plant (Kruskal–Wallis: *P* = 0.520–0.540; Wilcoxon: *P* = 0.178–0.904). Differences in alpha diversity were also not significant between geographic regions (Kruskal–Wallis: *P* = 0.150–0.300; Wilcoxon: *P* = 0.104–0.907). Similarly, for the secondary symbiont community, no significant differences in the alpha diversity were observed with respect to the host plant [Kruskal–Wallis: *P* = 0.170–0.410; Wilcoxon: *P* = 0.141–0.978 (Solanaceae: a higher Shannon index than the mean value across all groups, *P* = 0.024)] or geography [Kruskal–Wallis: *P* = 0.140–0.520; Wilcoxon: *P* = 0.127–0.954 (Xinjiang4: a higher Shannon index than the mean value across all groups, *P* = 0.029)].

**Fig. 3:**
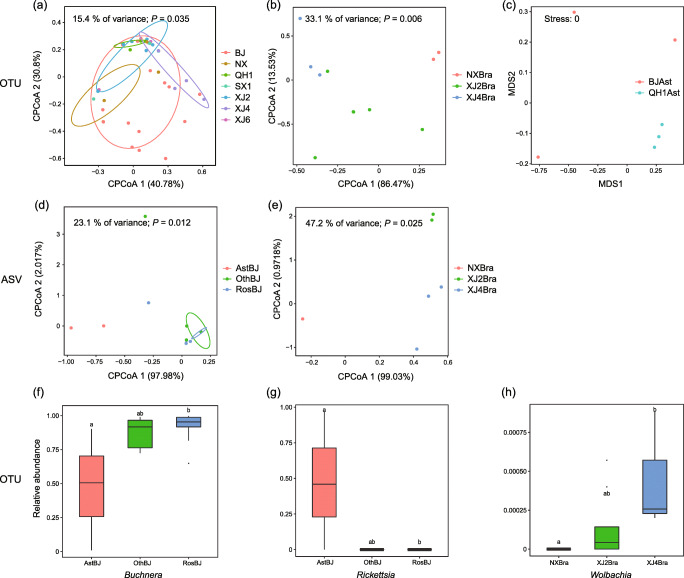
Variations observed in the symbiont communities associated with *Myzus persicae*. **a**–**e** Constrained principal coordinate analyses (cPCoA) (**a**, **b**, **d**, **e**) and nonmetric multidimensional scaling (NMDS) (**c**) of Bray–Curtis distances of symbiont communities at the OTU (**a**–**c**) and ASV (**d**, **e**) levels. **a** Seven geographic groups (*n* ≥ 3). **b**, **e** Three geographic groups on Brassicaceae (*n* ≥ 3). **c** Two geographic groups on Asteraceae (*n* ≥ 3). **d** Three host plant groups from Beijing (*n* ≥ 3). **f–h** Comparisons of relative abundances of *Buchnera* (**f**), *Rickettsia* (**g**), and *Wolbachia* (**h**) based on the OTU data. The different letters above boxes indicate significant differences (*P* < 0.05) following Fisher’s least significant difference (LSD) post hoc tests. Ast, Asteraceae; Bra, Brassicaceae; Ros, Rosaceae; Oth, other plant families; BJ, Beijing; NX, Ningxia; QH1, Qinghai1; SX1, Shaanxi1; XJ2, Xinjiang2; XJ4, Xinjiang4; XJ6, Xinjiang6

In the NMDS and cPCoA analyses of the Bray–Curtis dissimilarity of the symbiont community, there was no recognizable clustering of samples representing either different host plant families or geographic regions at both the OTU and ASV levels (NMDS: stress = 0.073–0.141 for OTU, ASV data were insufficient for NMDS; cPCoA: *P* = 0.077–0.680 for OTU, *P* = 0.180–0.860 for ASV; Fig. S2), except for the ordinations of seven geographic groups with a sample size ≥ 3 (OTU) (cPCoA: 15.4% of variance, *P* = 0.035; Fig. 3a), three geographic groups on Brassicaceae (OTU/ASV) (cPCoA: 33.1% of variance, *P* = 0.006 for OTU, Fig. 3b; cPCoA: 47.2% of variance, *P* = 0.025 for ASV, Fig. 3e), two geographic groups on Asteraceae (OTU) (NMDS: stress = 0; Fig. 3c), and three host plant groups from Beijing (ASV) (cPCoA: 23.1% of variance, *P* = 0.012; Fig. 3d). In general, ANOSIM and PERMANOVA revealed no significant differences in the symbiont community in response to the host plant or geography (*P* > 0.05, Table 2). The symbiont compositions were only observed to differ among the three host plant groups from Beijing (ANOSIM: *P* < 0.05 for ASV; PERMANOVA: *P* < 0.05 for OTU and ASV; Table 2) and among the three geographic groups on the plants of Brassicaceae (PERMANOVA: *P* < 0.05 for OTU and ASV; Table 2), which was consistent with the cPCoA results. The Kruskal–Wallis tests conducted on the OTU data set further showed that *Buchnera* (*P* = 0.049), *Rickettsia* (*P* = 0.037), and *Wolbachia* (*P* = 0.027) were present in significantly different relative abundances among these groups. The Asteraceae-feeding aphid populations in Beijing harbored a significantly low abundance of *Buchnera* (LSD: *P* < 0.05; Fig. 3f) but a high abundance of *Rickettsia* (LSD: *P* < 0.05; Fig. 3g). Within the three geographic aphid groups occurring on Brassicaceae, the relative abundance of *Wolbachia* in Xinjiang4 was statistically higher than that in Ningxia (LSD: *P* < 0.05; Fig. 3h).

**Table 2:** ANOSIM and PERMANOVA results for symbiont communities from different groups

Groups		Symbiont community				Secondary symbiont community			
		ANOSIM (*R*, *P*)		PERMANOVA (*R*^2^, *P*)		ANOSIM (*R*, *P*)		PERMANOVA (*R*^2^, *P*)	
		OTU	ASV	OTU	ASV	OTU	ASV	OTU	ASV
Host plant	All 16 groups	0.008, 0.418	− 0.036, 0.773	0.148, 0.528	0.146, 0.454	0.010, 0.390	0.076, 0.168	0.215, 0.437	0.415, 0.095
	7 groups (*n* ≥ 3)	0.011, 0.371	− 0.008, 0.543	0.093, 0.220	0.118, 0.092	− 0.008, 0.537	0.059, 0.208	0.091, 0.570	0.329, 0.110
	3 groups from Beijing (*n* ≥ 3)	0.166, 0.062	0.196, 0.038*	0.212, 0.040*	0.326, 0.013*	0.033, 0.319	− 0.005, 0.453	0.121, 0.405	0.137, 0.441
	Samples on primary and secondary host plants	− 0.019, 0.586	− 0.055, 0.744	0.006, 0.667	0.003, 0.830	− 0.010, 0.516	− 0.047, 0.590	0.004, 0.908	0.001, 0.924
Geography	All 30 groups	− 0.079, 0.849	− 0.131, 0.961	0.321, 0.487	0.227, 0.814	− 0.035, 0.622	− 0.145, 0.841	0.410, 0.193	0.489, 0.456
	7 groups (*n* ≥ 3)	− 0.112, 0.951	− 0.096, 0.908	0.154, 0.111	0.143, 0.215	− 0.099, 0.908	− 0.188, 0.969	0.176, 0.159	0.269, 0.497
	2 groups on Asteraceae (*n* ≥ 3)	0.330, 0.200	0.333, 1.000	0.396, 0.400	0.438, 0.400	0.111, 0.400	–	0.310, 0.400	0.137, 0.441
	3 groups on Brassicaceae (*n* ≥ 3)	0.177, 0.096	0.197, 0.088	0.418, 0.022*	0.508, 0.027*	0.202, 0.166	0, 1.000	0.481, 0.116	–

*P values < 0.05

*OTU* operational taxonomic unit, *ASV* amplicon sequence variant

When investigating the beta-diversity pattern of the secondary symbiont community, only cPCoA analyses of the OTU data set were performed, as the OTU data were insufficient for NMDS, and the ASV data were insufficient for both ordination analyses. Constrained PCoA plots of the OTU data showed that the secondary symbiont compositions did not change significantly with the host plant or geography (*P* = 0.170–0.630; Fig. S3). ANOSIM and PERMANOVA performed on both OTU and ASV data sets suggested no impacts of host plant or geography on the secondary symbiont community structure (*P* > 0.05, Table 2).

Furthermore, at the OTU level, no correlation between geographic distances and Bray–Curtis dissimilarities of symbiont (*r* = − 0.014, *P* = 0.589) or secondary symbiont communities (*r* = 0.009, *P* = 0.432) was observed in the Mantel tests.

## Discussion

In the present study, the microbiota of *M. persicae* was dominated by relatively few bacteria, which were primarily heritable symbionts. As in most other aphid species [22–25, 44], *B. aphidicola* was the predominant symbiont in *M. persicae*, being present in all samples and showing the highest average relative abundance*.* This is due to the fact that *B. aphidicola* is required for aphid development and reproduction [3]; it is strictly vertically transmitted and has undergone long-term parallel evolution with its aphid hosts [5]. Apart from four secondary symbionts that have been reported in previous studies (i.e., *H. defensa*, *R. insecticola*, *S. symbiotica*, and *Wolbachia*) [27, 28], *Rickettsia*, *Arsenophonus*, and *Spiroplasma* were also detected for the first time in the present study. Gallo-Franco et al. [29] did not report any secondary symbionts in their examined *M. persicae* samples, which may have been due to improper processing of the data. In their study, many OTUs were only annotated to bacterial families, and OTUs with relative abundances lower than 1% were ignored. However, secondary symbionts may occur at very low titers in their host aphids (e.g., Xu et al. [25]).

It is worth noting that two *M. persicae* samples on *C. sonchifolium* (Asteraceae) harbored a high abundance of *Rickettsia*. In most cases, the relative abundance of *Rickettsia* in *C. sonchifolium*-feeding aphid populations was greater than that of *Buchnera* (Figs. 2a and S1), which also led to the significantly higher relative abundance of *Rickettsia* in the Asteraceae-feeding populations in Beijing. Sakurai et al. [45] observed a significantly suppressed population density of *Buchnera* in the presence of *Rickettsia* in *Acyrthosiphon pisum*, which may also be the case for the two *M. persicae* samples occurring on *C. sonchifolium* assayed in the present study. Łukasik et al. [12] demonstrated that *Rickettsia* in the pea aphid could confer resistance against entomopathogens. In this study, the high abundance of *Rickettsia* was host-plant specific, suggesting that *Rickettsia* may have positive effects on the *C. sonchifolium*-feeding aphid populations*.* Further experiments are required to elucidate the potential role of *Rickettsia* in the adaptation of *M. persicae* to *C. sonchifolium*.

*Wolbachia* was the most prevalent secondary symbiont of *M. persicae*, a symbiont that is widespread in arthropods [46] but was previously considered to be rare in aphids [47]. Augustinos et al. [48] detected *Wolbachia* in 8.7% of European aphids and speculated that its occurrence in aphids was underestimated. Some studies have shown that *Wolbachia* is much more prevalent in aphids than previously believed [23, 44, 49, 50]. The high occurrence of *Wolbachia* indicates that it may play important roles in aphids. *Wolbachia* is well known for manipulating the reproduction of its arthropod hosts [51], although its exact role in aphids remains ambiguous. *Wolbachia* was suggested to be correlated with the prevalence of asexual lineages in some aphid species [49, 52], but this possibility has not been confirmed. De Clerck et al*.* [53] proposed that *Wolbachia* is involved in the nutrient production in *Pentalonia nigronervosa*, although this theory is controversial [54]. Therefore, the effects of *Wolbachia* in aphids should be investigated further.

*S. symbiotica*, *R. insecticola*, and *H. defensa* were reported to be present in *M. persicae* with very low prevalence (each symbiont: 1/50) in a study of Henry et al. [27] and were also detected in our samples at low infection frequencies*.* The relative abundances of *S. symbiotica* and *R. insecticola* were extremely low (< 0.02% across infected samples). *H. defensa* was only detected in four of 92 samples; however, it showed an average relative abundance of 3.45% across infected samples (Table 1). Because *H. defensa* can confer resistance to parasitoid attack [11], relatively high abundance of *H. defensa* in infected samples may indicate its importance as a defensive symbiont of aphids. In experimental populations of the pea aphid, the dynamics of *H. defensa* infection were observed to be related to the pressure from parasitoid wasps [55]. Therefore, low parasitism pressure may account for the low incidence of *H. defensa* observed in our study.

*Arsenophonus* and *Spiroplasma* were also reported here for the first time in the present study. *Arsenophonus* was harbored by 15 samples at low abundance. *Arsenophonus* has been identified in many insect groups [56] and in recent years has been shown to be a widespread secondary symbiont in aphids [23, 25, 57] that can enhance aphid performance on specific host plants [58]. However, *Arsenophonus* was not detected in previous studies of *M. persicae* [29, 57]. Although *Arsenophonus* may have truly been absent from these previously examined samples, the possibility that its absence was caused by false negatives in their screenings (diagnostic PCRs and improper data analyses) cannot be discarded. Among all seven secondary symbionts associated with *M. persicae*, *Spiroplasma* showed the lowest infection frequency and relative abundance across all samples. Its relative abundance across infected samples was also extremely low (0.006%). To date, *Spiroplasma* has rarely been detected in aphids [25, 59]. In *A. pisum*, *Spiroplasma* was shown to be abundant [60], and it can reduce male production [14] and provide resistance against natural enemies [12, 61]. However, in other aphids, its occurrence may have been overlooked probably due to its low titer. High-throughput sequencing can be helpful for *Spiroplasma* detection (e.g., Xu et al. [25] and the present study), and quantitative real-time PCR and fluorescence in situ hybridization (FISH) are certainly needed for its further precise detection.

The ordination analyses and statistical tests based on both the OTU and ASV data sets suggested that host plant and geography contributed little to the community structures of symbionts and secondary symbionts in *M. persicae*. Many studies on polyphagous aphids have revealed a crucial role of host plants in shaping symbiont composition [20, 25, 27]. However, in our present study, the secondary symbiont community did not change with the host plant, and symbiont community variation with respect to the host plant was only observed among the three Beijing groups on different plants (Fig. 3d, Table 2). As mentioned above, the high abundance of *Rickettsia* in *C. sonchifolium*-feeding populations contributed to these differences. Some studies have also shown geographical variation in aphid symbiont flora [17, 19, 44]. However, we observed no correlation between the spatial distances of sampling sites and the symbiont or secondary symbiont community structures. We also detected no significant community differences over geographical space, except in the three Brassicaceae-feeding symbiont community groups from different regions, which was supported by both ordination plots and statistical tests at the OTU and ASV levels (Fig. 3b, e, Table 2). Previous studies on the polyphagous *Aphis gossypii* have reported that geography has an effect on the symbiont communities of aphid populations that colonize a limited number of host plants [19, 25, 44]. However, spatial variation in the two geographic groups occurring on Asteraceae was only observed in the NMDS plot (Fig. 3c), which may be due to the two *C. sonchifolium*-feeding samples from Beijing, and this variation was not corroborated by statistical tests (Table 2). Therefore, it is also possible that the symbiont variation in Brassicaceae-feeding groups was caused by factors other than geography.

The results of the present study revealed no significant impacts of host plant or geography on the symbiont flora associated with *M. persicae.* Aphids and their bacterial partners are involved in a dynamic ecosystem. The prevalence and relative abundance of specific symbionts may be related to other ecological factors and selection pressures from the varying environment, such as ant attendance [27] and exposure to natural enemies [55].

*M. persicae* is a heteroecious holocyclic species, with anholocyclic populations occurring in warmer climates [26]. This species moves seasonally between primary and secondary host plants and migrates among diverse secondary hosts. We investigated the population genetic structure of *M. persicae* using the COI gene (detailed methods are provided in the Supplementary Methods). Among all 16 haplotypes identified, H1 was the most common haplotype and was shared by different populations across most sampling regions (Fig. 1). Furthermore, no significant isolation by distance (IBD) relationship was detected between population genetic differentiation and geographic distance (*r* = 0.142, *P* = 0.067). The widespread sharing of haplotype H1 and the absence of an IBD pattern suggests that *M. persicae* in China have undergone repeated migrations between populations. Because the host plants of *M. persicae* are primarily crops and ornamental plants, human activities may have greatly facilitated its dispersal. It appears that the absence of distinct patterns of symbiont community is normal within such a large panmictic population of *M. persicae*. In addition, secondary symbionts can be horizontally transmitted between aphids during sexual reproduction [62] as well as via the host plant [63] or parasitoids [64]. Same phylotype (OTU) of secondary symbionts was shared by different populations of *M. persicae*, suggesting that horizontal transmission may have occurred. Frequent migrations between different populations, coupled with intraspecific horizontal transmission of secondary symbionts, may have caused the absence of distinct symbiont community patterns in *M. persicae*.

## Conclusions

Characterizing symbiont communities is fundamental to understanding host-symbiont systems. In the present study, based on a broad sampling, we provide the first comprehensive survey of symbiont diversity within *M. persicae*, a representative polyphagous aphid species. Heritable bacterial symbionts comprised the major components of the *M. persicae* microbiome*.* The symbiont compositions were not significantly altered across natural aphid populations. Specific biological characteristics of *M. persicae* and human activities may together have contributed to the absence of distinct patterns in symbiont community. Thus, the results of our study highlight the importance of the host and environment in microbiome assemblage.

## Electronic supplementary material

ESM 1 (DOCX 6620 kb)
