# Supplementary material for: Diversity of bacterial symbionts associated with Myzus persicae (Sulzer) (Hemiptera: Aphididae: Aphidinae) revealed by 16S rRNA Illumina sequencing
Source: Microb Ecol. 2020 Oct 17;81(3):784–94. doi: 10.1007/s00248-020-01622-6 (PMC7982390; doi:10.1007/s00248-020-01622-6)
Supplement: Supplementary file 1 — (DOCX 6620 kb) [file 248_2020_1622_MOESM1_ESM.docx]

**Electronic Supplementary Material**

**Diversity of bacterial symbionts associated with *Myzus persicae* (Sulzer) (Hemiptera: Aphididae: Aphidinae) revealed by 16S rRNA Illumina sequencing**

Shifen Xu^1,2^, Liyun Jiang^1^, Gexia Qiao^1,2^, Jing Chen^1^

^1^ Key Laboratory of Zoological Systematics and Evolution, Institute of Zoology, Chinese Academy of Sciences, Beijing 100101, China

^2^ College of Life Sciences, University of Chinese Academy of Sciences, Beijing 100049, China

**Corresponding authors**

Gexia Qiao, [qiaogx@ioz.ac.cn](mailto:qiaogx@ioz.ac.cn); Jing Chen, chenjing@ioz.ac.cn.

**Supplementary Methods**

**Methods for Haplotype and Isolation by Distance Analyses**

The haplotypes in *Myzus persicae* populations were estimated with DNASP v6.12.03 [1] based on COI sequences. The spatial genetic structure was then investigated by testing for the pattern of isolation by distance (IBD). Under geographically restricted dispersal, limited gene flow will result in a positive correlation between genetic differentiation between populations and the geographic distance separating them [2]. To assess IBD, a Mantel test was performed using the function *mantel* in the *vegan* package [3]. Pairwise *F*_ST_ values and geographic distances among populations were calculated using Arlequin v3.5.2.2 [4] and Geographic Distance Matrix Generator v1.2.3 [5], respectively.

**References**

1. Rozas J, Ferrer-Mata A, Sánchez-DelBarrio JC, Guirao-Rico S, Librado P, Ramos-Onsins SE, Sánchez-Gracia A (2017) DnaSP 6: DNA sequence polymorphism analysis of large data sets. Mol Biol Evol 34:3299–3302. https://doi.org/10.1093/molbev/msx248
2. Wright S (1943) Isolation by distance. Genetics 28:14–138.
3. Oksanen J, Blanchet FG, Friendly M et al (2018) Package ‘vegan’, community ecology package. Version 2.5-2. https://github.com/vegandevs/vegan. Accessed 24 Aug 2019
4. Excoffier L, Lischer HEL (2010) Arlequin suite ver 3.5: a new series of programs to perform population genetics analyses under Linux and Windows. Mol Ecol Resour 10:564–567. https://doi.org/10.1111/j.1755-0998.2010.02847.x
5. Ersts PJ (2020) Geographic Distance Matrix Generator v1.2.3. American Museum of Natural History, Center for Biodiversity and Conservation. http://biodiversityinformatics.amnh.org/open_source/gdmg. Accessed 13 Jan 2020

**Table S1.** Voucher information and GenBank accession numbers of the *Myzus persicae* samples used in the present study.

| Sample ID | Date | Host plant | Location | COI |
| --- | --- | --- | --- | --- |
| M10499 | 10 May 2016 | *Crepidiastrum sonchifolium* | Beijing, China | MT127004 |
| M13463 | 27 Apr. 2004 | *Kochia scoparia* | Beijing, China | MT127005 |
| M13541 | 4 Jul. 2002 | *Amygdalus persica* | Nyingchi, Tibet, China | MT127006 |
| M13585 | 2 Aug. 2002 | *Fagraea ceilanica* | Xining, Qinghai, China | MT127007 |
| M13596 | 3 Aug. 2002 | Brassicaceae | Menyuan, Qinghai, China | MT127008 |
| M13691 | 21 Aug. 2002 | *Celosia cristata* | Burqin, Xinjiang, China | MT127009 |
| M13693 | 21 Aug. 2002 | *Zinnia elegans* | Burqin, Xinjiang, China | MT127010 |
| M13919 | 18 Sep. 2002 | *Solanum tuberosum* | Aketao, Xinjiang, China | MT127011 |
| M13930 | 18 Sep. 2002 | *Ipomoea nil* | Aketao, Xinjiang, China | MT127012 |
| M13989 | 24 Sep. 2002 | *Alcea rosea* | Pishan, Xinjiang, China | MT127013 |
| M13995 | 25 Sep. 2002 | *Amygdalus persica* | Pishan, Xinjiang, China | MT127014 |
| M13996 | 25 Sep. 2002 | *Armeniaca vulgaris* | Pishan, Xinjiang, China | MT127015 |
| M14027 | 30 Sep. 2002 | *Amygdalus persica* | Ruoqiang, Xinjiang, China | MT127016 |
| M14056 | 3 Oct. 2002 | *Ulmus pumila* | Delingha, Qinghai, China | MT127017 |
| M14111 | 4 Oct. 2002 | *Lactuca sativa* | Ulan, Qinghai, China | MT127018 |
| M14115 | 4 Oct. 2002 | *Coriandrum sativum* | Ulan, Qinghai, China | MT127019 |
| M14116 | 4 Oct. 2002 | Asteraceae | Ulan, Qinghai, China | MT127020 |
| M14117 | 4 Oct. 2002 | Asteraceae | Ulan, Qinghai, China | MT127021 |
| M14119 | 4 Oct. 2002 | *Alcea rosea* | Ulan, Qinghai, China | MT127022 |
| M14127 | 9 Oct. 2002 | *Alcea rosea* | Jingbian, Shaanxi, China | MT127023 |
| M14128 | 9 Oct. 2002 | *Alcea rosea* | Jingbian, Shaanxi, China | MT127024 |
| M15162 | 22 May 2004 | *Artemisia* sp. | Beijing, China | MT127025 |
| M15248 | 20 May 2004 | Rosaceae | Beijing, China | MT127026 |
| M15254 | 20 May 2004 | *Ipomoea nil* | Beijing, China | MT127027 |
| M16122 | 2 May 2005 | *Amygdalus persica* | Dongguang, Hebei, China | MT127028 |
| M16439 | 16 Jul. 2005 | *Amygdalus persica* | Zayu, Tibet, China | MT127029 |
| M16542 | 25 Apr. 2006 | *Amygdalus persica* | Beijing, China | MT127030 |
| M16549 | 29 Apr. 2006 | *Amygdalus persica* | Beijing, China | MT127031 |
| M16597 | 24 Aug. 2005 | *Brassica rapa* var. *glabra* | Habahe, Xinjiang, China | MT127032 |
| M16599 | 24 Aug. 2005 | *Brassica oleracea* var. *capitata* | Habahe, Xinjiang, China | MT127033 |
| M16615 | 22 Aug. 2005 | *Raphanus sativus* | Fuyun, Xinjiang, China | MT127034 |
| M16617 | 22 Aug. 2005 | *Brassica oleracea* var. *capitata* | Fuyun, Xinjiang, China | MT127035 |
| M16624 | 22 Aug. 2005 | *Brassica rapa* var. *glabra* | Fuyun, Xinjiang, China | MT127036 |
| M16634 | 21 Aug. 2005 | *Brassica oleracea* var. *capitata* | Qinghe, Xinjiang, China | MT127037 |
| M16652 | 18 Aug. 2005 | *Brassica rapa* var. *glabra* | Fuhai, Xinjiang, China | MT127038 |
| M16653 | 18 Aug. 2005 | *Raphanus sativus* | Fuhai, Xinjiang, China | MT127039 |
| M16659 | 18 Aug. 2005 | *Brassica oleracea* var. *capitata* | Fuhai, Xinjiang, China | MT127040 |
| M16660 | 18 Aug. 2005 | *Solanum melongena* | Fuhai, Xinjiang, China | MT127041 |
| M16670 | 16 Aug. 2005 | *Brassica rapa* var. *glabra* | Emin, Xinjiang, China | MT127042 |
| M16678 | 14 Aug. 2005 | *Lycopersicon esculentum* | Toli, Xinjiang, China | MT127043 |
| M16683 | 14 Aug. 2005 | *Brassica oleracea* var. *capitata* | Toli, Xinjiang, China | MT127044 |
| M16688 | 13 Aug. 2005 | Solanaceae | Yumin, Xinjiang, China | MT127045 |
| M16690 | 13 Aug. 2005 | *Raphanus sativus* | Yumin, Xinjiang, China | MT127046 |
| M16792 | 2 Aug. 2005 | *Solanum melongena* | Gongliu, Xinjiang, China | MT127047 |
| M16902 | 22 Jul. 2005 | *Brassica rapa* var. *oleifera* | Qira, Xinjiang, China | MT127048 |
| M17002 | 22 Mar. 2005 | *Brassica rapa* var. *oleifera* | Yang County, Shaanxi, China | MT127049 |
| M17036 | 29 Mar. 2005 | *Raphanus sativus* | Yanbian, Sichuan, China | MT127050 |
| M17208 | 25 Apr. 2005 | *Armeniaca vulgaris* | Miyi, Sichuan, China | MT127051 |
| M17750 | 6 Aug. 2005 | *Nicotiana tabacum* | Fujin, Heilongjiang, China | MT127052 |
| M17751 | 6 Aug. 2005 | *Nicotiana tabacum* | Fujin, Heilongjiang, China | MT127053 |
| M18123 | 13 Oct. 2005 | *Arabidopsis thaliana* | Beijing, China | MT127054 |
| M18278 | 10 Aug. 2005 | *Tagetes erecta* | Golmud, Qinghai, China | MT127055 |
| M18282 | 10 Aug. 2005 | *Tagetes erecta* | Golmud, Qinghai, China | MT127056 |
| M18712 | 8 May 2006 | *Punica granatum* | Jianchuan, Yunnan, China | MT127057 |
| M18835 | 14 May 2006 | *Prunus cerasifera* f. *atropurpurea* | Beijing, China | MT127058 |
| M18838 | 14 May 2006 | *Hibiscus* *syriacus* | Beijing, China | MT127059 |
| M19550 | 20 Apr. 2007 | *Spinacia oleracea* | Rizhao, Shandong, China | MT127060 |
| M19555 | 21 Apr. 2007 | *Amygdalus persica* | Rizhao, Shandong, China | MT127061 |
| M20251 | 29 Jul. 2007 | Asteraceae | Yuexi, Anhui, China | MT127062 |
| M20519 | 27 Jul. 2007 | *Bougainvillea spectabilis* | Puer, Yunnan, China | MT127063 |
| M21102 | 21 Jun. 2008 | *Dahlia pinnata* | Jingyuan, Ningxia, China | MT127064 |
| M21127 | 24 Jun. 2008 | *Brassica oleracea* var. *capitata* | Jingyuan, Ningxia, China | MT127065 |
| M21128 | 24 Jun. 2008 | *Brassica oleracea* var. *capitata* | Jingyuan, Ningxia, China | MT127066 |
| M21146 | 27 Jun. 2008 | *Brassica oleracea* var. *albiflora* | Jingyuan, Ningxia, China | MT127067 |
| M21147 | 27 Jun. 2008 | *Brassica oleracea* var. *albiflora* | Jingyuan, Ningxia, China | MT127068 |
| M21551 | 29 Jun. 2008 | *Chenopodium glaucum* | Jingyuan, Ningxia, China | MT127069 |
| M21680 | 4 Jul. 2008 | *Solanum tuberosum* | Jingyuan, Ningxia, China | MT127070 |
| M22883 | 13 May 2009 | *Crepidiastrum sonchifolium* | Beijing, China | MT127071 |
| M23037 | 1 Jun. 2009 | *Foeniculum vulgare* | Tianshui, Gansu, China | MT127072 |
| M23083 | 3 Jun. 2009 | *Helianthus annuus* | Tianshui, Gansu, China | MT127073 |
| M23189 | 23 Jun. 2009 | *Gardenia jasminoides* | Beijing, China | MT127074 |
| M23996 | 26 Nov. 2009 | *Bougainvillea spectabilis* | Puer, Yunnan, China | MT127075 |
| M24103 | 14 Dec. 2009 | *Forsythia suspensa* | Longling, Yunnan, China | MT127076 |
| M24385 | 16 May 2010 | *Amygdalus persica* | Beijing, China | MT127077 |
| M24438 | 22 May 2010 | *Ipomoea nil* | Ankang, Shaanxi, China | MT127078 |
| M24465 | 18 May 2010 | *Amygdalus persica* | Beijing, China | MT127079 |
| M25146 | 4 Aug. 2010 | *Raphanus sativus* | Alxa, Inner Mongolia, China | MT127080 |
| M25201 | 17 Aug. 2010 | *Solanum melongena* | Alxa, Inner Mongolia, China | MT127081 |
| M25430 | 16 Nov. 2010 | Solanaceae | Jiayi, Taiwan, China | MT127082 |
| M26240 | 17 Nov. 2010 | *Ipomoea batatas* | Chongzuo, Guangxi, China | MT127083 |
| M27456 | 11 Mar. 2012 | *Nicotiana tabacum* | Wuzhishan, Hainan, China | MT127084 |
| M27494 | 13 May 2012 | *Amygdalus persica* | Beijing, China | MT127085 |
| M27624 | 20 May 2012 | *Amygdalus davidiana* | Huhhot, Inner Mongolia, China | MT127086 |
| M28399 | 17 May 2013 | *Amygdalus persica* | Beijing, China | MT127087 |
| M28414 | 19 May 2013 | *Zanthoxylum bungeanum* | Beijing, China | MT127088 |
| M28420 | 19 May 2013 | *Amygdalus persica* var. *compressa* | Beijing, China | MT127089 |
| M28421 | 19 May 2013 | *Amygdalus persica* var. *compressa* | Beijing, China | MT127090 |
| M36156 | 17 Nov. 2015 | *Capsicum annuum* | Beijing, China | MT127091 |
| M36554 | 24 Nov. 2015 | *Cyphomandra betacea* | Nantou, Taiwan, China | MT127092 |
| M37004 | 20 May 2016 | *Solanum nigrum* | Beijing, China | MT127093 |
| M37103 | 12 May 2016 | *Amygdalus persica* | Beijing, China | MT127094 |
| Y9306 | 26 Jun. 2013 | *Nicotiana tabacum* | Yangling, Shaanxi, China | MT127095 |

**Table S2.** Grouping information for the *Myzus persicae* samples used in the present study.

| Host plant | Number of samples | Sample ID |
| --- | --- | --- |
| Amaranthaceae (Ama) | 1 | M13691 |
| Apiaceae (Api) | 2 | M14115, M23037 |
| Asteraceae (Ast) | 12 | M10499, M13693, M14111, M14116, M14117, M15162, M18278, M18282, M20251, M21102, M22883, M23083 |
| Brassicaceae (Bra) | 22 | M13596, M16597, M16599, M16615, M16617, M16624, M16634, M16652, M16653, M16659, M16670, M16683, M16690, M16902, M17002, M17036, M18123, M21127, M21128, M21146, M21147, M25146 |
| Chenopodiaceae (Che) | 3 | M13463, M19550, M21551 |
| Convolvulaceae (Con) | 4 | M13930, M15254, M24438, M26240 |
| Loganiaceae (Log) | 1 | M13585 |
| Lythraceae (Lyt) | 1 | M18712 |
| Malvaceae (Mal) | 5 | M13989, M14119, M14127, M14128, M18838 |
| Nyctaginaceae (Nyc) | 2 | M20519, M23996 |
| Oleaceae (Ole) | 1 | M24103 |
| Rosaceae (Ros) | 20 | M13541, M13995, M13996, M14027, M15248, M16122, M16439, M16542, M16549, M17208, M18835, M19555, M24385, M24465, M27494, M27624, M28399, M28420, M28421, M37103 |
| Rubiaceae (Rub) | 1 | M23189 |
| Rutaceae (Rut) | 1 | M28414 |
| Solanaceae (Sol) | 15 | M13919, M16660, M16678, M16688, M16792, M17750, M17751, M21680, M25201, M25430, M27456, M36156, M36554, M37004, Y9306 |
| Ulmaceae (Ulm) | 1 | M14056 |
| Geographic region | Number of samples | Sample ID |
| Anhui (AH) | 1 | M20251 |
| Beijing (BJ) | 22 | M10499, M13463, M15162, M15248, M15254, M16542, M16549, M18123, M18835, M18838, M22883, M23189, M24385, M24465, M27494, M28399, M28414, M28420, M28421, M36156, M37004, M37103 |
| Gansu (GS) | 2 | M23037, M23083 |
| Guangxi (GX) | 1 | M26240 |
| Hainan (HN) | 1 | M27456 |
| Hebei (HB) | 1 | M16122 |
| Heilongjiang (HLJ) | 2 | M17750, M17751 |
| Inner Mongolia 1 (IM1) | 2 | M25146, M25201 |
| Inner Mongolia 2 (IM2) | 1 | M27624 |
| Ningxia (NX) | 7 | M21102, M21127, M21128, M21146, M21147, M21551, M21680 |
| Qinghai1 (QH1) | 6 | M14056, M14111, M14115, M14116, M14117, M14119 |
| Qinghai2 (QH2) | 2 | M18278, M18282 |
| Qinghai3 (QH3) | 2 | M13585, M13596 |
| Shaanxi1 (SX1) | 3 | M17002, M24438, Y9306 |
| Shaanxi2 (SX2) | 2 | M14127, M14128 |
| Shandong (SD) | 2 | M19550, M19555 |
| Sichuan (SC) | 2 | M17036, M17208 |
| Taiwan (TW) | 2 | M25430, M36554 |
| Tibet (TB) | 2 | M13541, M16439 |
| Xinjiang1 (XJ1) | 2 | M13919, M13930 |
| Xinjiang2 (XJ2) | 11 | M13691, M13693, M16597, M16599, M16615, M16617, M16624, M16652, M16653, M16659, M16660 |
| Xinjiang3 (XJ3) | 1 | M16902 |
| Xinjiang4 (XJ4) | 5 | M16670, M16678, M16683, M16688, M16690 |
| Xinjiang5 (XJ5) | 1 | M16792 |
| Xinjiang6 (XJ6) | 3 | M13989, M13995, M13996 |
| Xinjiang7 (XJ7) | 1 | M16634 |
| Xinjiang8 (XJ8) | 1 | M14027 |
| Yunnan1 (YN1) | 1 | M18712 |
| Yunnan2 (YN2) | 2 | M20519, M23996 |
| Yunnan3 (YN3) | 1 | M24103 |

**Table S3.** Relative abundances of the top 10 bacterial phyla, classes, orders, families and genera in *Myzus persicae.*

| Phylum (%) | Class (%) | Order (%) | Family (%) | Genus (%) |
| --- | --- | --- | --- | --- |
| Proteobacteria/96.83 | Gammaproteobacteria/92.93 | Enterobacteriales/91.37 | Enterobacteriaceae/91.37 | ***Buchnera*/90.77** |
| Actinobacteria/0.89 | Alphaproteobacteria/2.72 | Rickettsiales/1.61 | Rickettsiaceae/1.59 | ***Rickettsia*/1.59** |
| Bacteroidetes/0.85 | Betaproteobacteria/1.07 | Pseudomonadales/1.20 | Moraxellaceae/1.03 | *Acinetobacter*/0.98 |
| Firmicutes/0.74 | Actinobacteria/0.78 | Burkholderiales/1.00 | Oxalobacteraceae/0.61 | *Duganella*/0.56 |
| Acidobacteria/0.11 | Bacteroidia/0.50 | Rhizobiales/0.56 | Brucellaceae/0.36 | *Brucella*/0.36 |
| Chloroflexi/0.10 | Clostridia/0.36 | Bacteroidales/0.50 | Corynebacteriaceae/0.28 | *Escherichia-Shigella*/0.31 |
| Deinococcus-Thermus/0.08 | Bacilli/0.32 | Clostridiales/0.36 | Comamonadaceae/0.28 | *Corynebacterium_1*/0.27 |
| Thermotogae/0.08 | Sphingobacteriia/0.17 | Corynebacteriales/0.30 | Ruminococcaceae/0.22 | *Pseudomonas*/0.17 |
| Nitrospirae/0.06 | Deltaproteobacteria/0.10 | Bacillales/0.23 | Prevotellaceae/0.18 | ***Hamiltonella*/0.15** |
| Gemmatimonadetes/0.05 | Flavobacteriia/0.09 | Micrococcales/0.20 | Pseudomonadaceae/0.17 | *Sphingomonas*/0.14 |

Symbionts are indicated in bold.

**Table S4.** Alpha diversity of the bacterial and symbiont communities from each sample.

| Sample ID | Bacterial community | | | Symbiont community | | | Secondary symbiont community | | |
| --- | --- | --- | --- | --- | --- | --- | --- | --- | --- |
|  | Number of OTUs | Shannon | Simpson | Number of OTUs | Shannon | Simpson | Number of OTUs | Shannon | Simpson |
| M10499 | 207 | 0.263 | 0.938 | 8 | 0.058 | 0.981 | 2 | 0 | 1 |
| M13463 | 235 | 1.378 | 0.585 | 11 | 0.071 | 0.981 | 0 | 0 | 0 |
| M13541 | 209 | 0.342 | 0.921 | 9 | 0.025 | 0.994 | 1 | 0 | 1 |
| M13585 | 148 | 0.386 | 0.903 | 11 | 0.063 | 0.983 | 2 | 0.562 | 0.625 |
| M13596 | 288 | 0.580 | 0.859 | 9 | 0.026 | 0.994 | 1 | 0 | 1 |
| M13691 | 291 | 0.568 | 0.866 | 13 | 0.067 | 0.982 | 2 | 0.377 | 0.781 |
| M13693 | 163 | 0.302 | 0.928 | 11 | 0.064 | 0.983 | 2 | 0.673 | 0.520 |
| M13919 | 73 | 0.097 | 0.977 | 10 | 0.045 | 0.988 | 3 | 1.040 | 0.375 |
| M13930 | 146 | 0.217 | 0.949 | 13 | 0.071 | 0.981 | 1 | 0 | 1 |
| M13989 | 157 | 0.291 | 0.929 | 10 | 0.068 | 0.982 | 1 | 0 | 1 |
| M13995 | 174 | 0.273 | 0.937 | 12 | 0.041 | 0.990 | 3 | 0.703 | 0.602 |
| M13996 | 231 | 0.437 | 0.899 | 10 | 0.042 | 0.990 | 1 | 0 | 1 |
| M14027 | 183 | 0.349 | 0.918 | 12 | 0.071 | 0.981 | 1 | 0 | 1 |
| M14056 | 182 | 0.349 | 0.917 | 11 | 0.077 | 0.979 | 1 | 0 | 1 |
| M14111 | 119 | 0.193 | 0.954 | 11 | 0.062 | 0.984 | 1 | 0 | 1 |
| M14115 | 224 | 0.362 | 0.916 | 10 | 0.055 | 0.986 | 1 | 0 | 1 |
| M14116 | 129 | 0.188 | 0.956 | 11 | 0.065 | 0.983 | 2 | 0.693 | 0.500 |
| M14117 | 136 | 0.182 | 0.958 | 12 | 0.065 | 0.983 | 1 | 0 | 1 |
| M14119 | 233 | 0.559 | 0.864 | 11 | 0.068 | 0.982 | 1 | 0 | 1 |
| M14127 | 136 | 0.216 | 0.950 | 6 | 0.038 | 0.990 | 0 | 0 | 0 |
| M14128 | 276 | 0.856 | 0.670 | 14 | 0.429 | 0.757 | 5 | 1.468 | 0.256 |
| M15162 | 148 | 0.596 | 0.809 | 8 | 0.031 | 0.992 | 1 | 0 | 1 |
| M15248 | 434 | 0.649 | 0.856 | 13 | 0.064 | 0.983 | 1 | 0 | 1 |
| M15254 | 152 | 0.217 | 0.950 | 11 | 0.064 | 0.983 | 1 | 0 | 1 |
| M16122 | 331 | 0.493 | 0.890 | 13 | 0.047 | 0.988 | 2 | 0.683 | 0.510 |
| M16439 | 398 | 0.801 | 0.818 | 9 | 0.023 | 0.995 | 1 | 0 | 1 |
| M16542 | 103 | 0.129 | 0.970 | 11 | 0.061 | 0.984 | 1 | 0 | 1 |
| M16549 | 179 | 1.160 | 0.658 | 8 | 0.068 | 0.982 | 1 | 0 | 1 |
| M16597 | 189 | 0.515 | 0.869 | 11 | 0.070 | 0.981 | 1 | 0 | 1 |
| M16599 | 270 | 0.405 | 0.907 | 12 | 0.069 | 0.981 | 1 | 0 | 1 |
| M16615 | 185 | 0.305 | 0.928 | 11 | 0.068 | 0.982 | 2 | 0.191 | 0.909 |
| M16617 | 261 | 0.692 | 0.832 | 10 | 0.061 | 0.984 | 0 | 0 | 0 |
| M16624 | 245 | 0.446 | 0.895 | 10 | 0.063 | 0.983 | 1 | 0 | 1 |
| M16634 | 152 | 0.376 | 0.906 | 10 | 0.065 | 0.983 | 0 | 0 | 0 |
| M16652 | 210 | 0.900 | 0.754 | 12 | 0.076 | 0.980 | 2 | 0.655 | 0.537 |
| M16653 | 147 | 0.122 | 0.974 | 9 | 0.044 | 0.989 | 0 | 0 | 0 |
| M16659 | 273 | 0.322 | 0.929 | 11 | 0.059 | 0.984 | 1 | 0 | 1 |
| M16660 | 219 | 0.271 | 0.940 | 11 | 0.038 | 0.990 | 2 | 0.500 | 0.680 |
| M16670 | 322 | 1.493 | 0.624 | 10 | 0.066 | 0.983 | 1 | 0 | 1 |
| M16678 | 325 | 0.719 | 0.830 | 14 | 0.064 | 0.983 | 3 | 0.796 | 0.551 |
| M16683 | 359 | 1.188 | 0.703 | 10 | 0.063 | 0.983 | 2 | 0.349 | 0.802 |
| M16688 | 754 | 3.185 | 0.308 | 12 | 0.093 | 0.975 | 4 | 0.673 | 0.612 |
| M16690 | 166 | 0.410 | 0.897 | 12 | 0.069 | 0.982 | 2 | 0.690 | 0.503 |
| M16792 | 232 | 0.539 | 0.867 | 10 | 0.050 | 0.987 | 2 | 0.325 | 0.820 |
| M16902 | 274 | 0.742 | 0.820 | 10 | 0.040 | 0.990 | 1 | 0 | 1 |
| M17002 | 194 | 0.573 | 0.845 | 11 | 0.064 | 0.983 | 1 | 0 | 1 |
| M17036 | 137 | 0.666 | 0.705 | 10 | 0.459 | 0.745 | 1 | 0 | 1 |
| M17208 | 163 | 0.303 | 0.927 | 9 | 0.029 | 0.993 | 1 | 0 | 1 |
| M17750 | 284 | 0.349 | 0.922 | 11 | 0.062 | 0.983 | 2 | 0.693 | 0.500 |
| M17751 | 277 | 0.828 | 0.791 | 12 | 0.082 | 0.978 | 2 | 0.637 | 0.556 |
| M18123 | 437 | 2.062 | 0.490 | 13 | 0.197 | 0.929 | 2 | 0.562 | 0.625 |
| M18278 | 319 | 0.637 | 0.847 | 12 | 0.061 | 0.984 | 1 | 0 | 1 |
| M18282 | 306 | 0.408 | 0.908 | 10 | 0.070 | 0.981 | 0 | 0 | 0 |
| M18712 | 74 | 0.080 | 0.983 | 9 | 0.024 | 0.994 | 3 | 1.099 | 0.333 |
| M18835 | 53 | 0.106 | 0.972 | 12 | 0.088 | 0.975 | 5 | 0.775 | 0.571 |
| M18838 | 454 | 0.829 | 0.811 | 11 | 0.070 | 0.981 | 1 | 0 | 1 |
| M19550 | 315 | 0.506 | 0.882 | 11 | 0.066 | 0.982 | 0 | 0 | 0 |
| M19555 | 258 | 0.443 | 0.895 | 11 | 0.064 | 0.983 | 1 | 0 | 1 |
| M20251 | 83 | 0.145 | 0.964 | 7 | 0.038 | 0.990 | 0 | 0 | 0 |
| M20519 | 229 | 0.321 | 0.925 | 9 | 0.067 | 0.982 | 0 | 0 | 0 |
| M21102 | 188 | 0.529 | 0.862 | 9 | 0.034 | 0.992 | 1 | 0 | 1 |
| M21127 | 207 | 0.409 | 0.901 | 11 | 0.073 | 0.980 | 0 | 0 | 0 |
| M21128 | 96 | 0.217 | 0.945 | 10 | 0.067 | 0.982 | 0 | 0 | 0 |
| M21146 | 279 | 0.708 | 0.825 | 10 | 0.074 | 0.980 | 0 | 0 | 0 |
| M21147 | 356 | 0.816 | 0.808 | 11 | 0.083 | 0.977 | 1 | 0 | 1 |
| M21551 | 205 | 1.783 | 0.472 | 10 | 0.113 | 0.965 | 2 | 0.224 | 0.889 |
| M21680 | 224 | 0.373 | 0.914 | 7 | 0.037 | 0.990 | 1 | 0 | 1 |
| M22883 | 181 | 0.980 | 0.463 | 8 | 0.717 | 0.498 | 1 | 0 | 1 |
| M23037 | 146 | 1.142 | 0.666 | 11 | 0.063 | 0.983 | 1 | 0 | 1 |
| M23083 | 276 | 1.026 | 0.667 | 16 | 0.709 | 0.726 | 8 | 1.057 | 0.416 |
| M23189 | 372 | 1.297 | 0.432 | 12 | 0.713 | 0.504 | 2 | 0.349 | 0.802 |
| M23996 | 205 | 1.010 | 0.723 | 8 | 0.033 | 0.992 | 1 | 0 | 1 |
| M24103 | 251 | 0.936 | 0.748 | 12 | 0.067 | 0.982 | 2 | 0.451 | 0.722 |
| M24385 | 144 | 0.395 | 0.896 | 10 | 0.061 | 0.984 | 0 | 0 | 0 |
| M24438 | 181 | 0.581 | 0.846 | 9 | 0.064 | 0.983 | 0 | 0 | 0 |
| M24465 | 115 | 0.209 | 0.950 | 10 | 0.059 | 0.984 | 0 | 0 | 0 |
| M25146 | 549 | 1.331 | 0.695 | 9 | 0.063 | 0.983 | 0 | 0 | 0 |
| M25201 | 234 | 0.296 | 0.935 | 11 | 0.045 | 0.989 | 2 | 0.108 | 0.956 |
| M25430 | 171 | 1.117 | 0.682 | 10 | 0.068 | 0.982 | 0 | 0 | 0 |
| M26240 | 207 | 1.365 | 0.569 | 13 | 0.074 | 0.980 | 2 | 0.349 | 0.802 |
| M27456 | 139 | 0.264 | 0.936 | 10 | 0.069 | 0.981 | 0 | 0 | 0 |
| M27494 | 56 | 0.103 | 0.975 | 12 | 0.073 | 0.981 | 2 | 0.500 | 0.680 |
| M27624 | 135 | 0.250 | 0.940 | 12 | 0.049 | 0.987 | 4 | 1.277 | 0.306 |
| M28399 | 313 | 0.399 | 0.912 | 9 | 0.066 | 0.982 | 0 | 0 | 0 |
| M28414 | 99 | 0.177 | 0.955 | 11 | 0.091 | 0.974 | 1 | 0 | 1 |
| M28420 | 391 | 0.716 | 0.837 | 10 | 0.075 | 0.980 | 1 | 0 | 1 |
| M28421 | 448 | 0.836 | 0.812 | 11 | 0.072 | 0.981 | 1 | 0 | 1 |
| M36156 | 248 | 0.616 | 0.822 | 15 | 0.376 | 0.871 | 7 | 1.017 | 0.438 |
| M36554 | 382 | 0.803 | 0.813 | 10 | 0.075 | 0.980 | 1 | 0 | 1 |
| M37004 | 364 | 1.871 | 0.548 | 10 | 0.072 | 0.981 | 2 | 0.637 | 0.556 |
| M37103 | 463 | 2.392 | 0.415 | 12 | 0.098 | 0.974 | 2 | 0.577 | 0.611 |
| Y9306 | 203 | 0.233 | 0.948 | 10 | 0.068 | 0.982 | 0 | 0 | 0 |
| Mean | 235 | 0.636 | 0.832 | 10 | 0.096 | 0.963 | 1 | 0.225 | 0.645 |


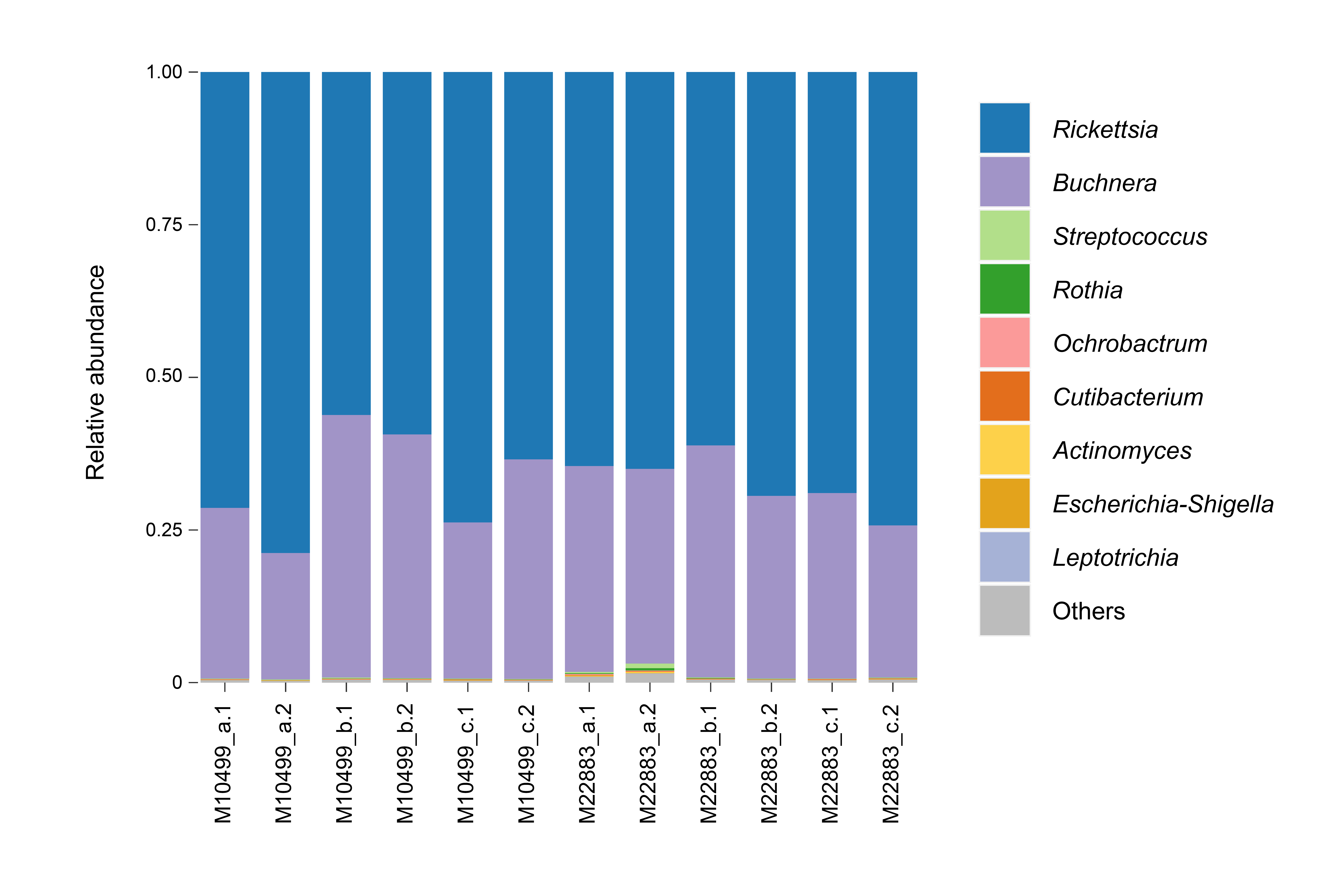


**Fig. S1** Bar plots of the bacterial communities determined using three biological replicates (i.e., three aphid individuals from one colony; indicated by “a”, “b” and “c”) and two PCR replicates (indicated by “1” and “2”) of two *Myzus persicae* samples feeding on *Crepidiastrum sonchifolium.*





**Fig. S2** No significant variation observed in the symbiont communities associated with *Myzus persicae*. **a**–**g** Nonmetric multidimensional scaling (NMDS) of Bray−Curtis distances at the OTU level. **h**–**o** Constrained principal coordinate analyses (cPCoA) of Bray−Curtis distances at the OTU (**h**–**k**) and ASV (**l**–**o**) levels. **a**, **h**, **l** All 16 host plant groups. **b**, **i**, **m** Seven host plant groups (n≥ 3). **c**, **j** Three host plant groups from Beijing (n ≥ 3). **d** Samples on primary and secondary host plants. **e**, **k**, **n** All 30 geographic groups. **f**, **o** Seven geographic groups (n ≥ 3). **g** Three geographic groups on Brassicaceae (n ≥ 3). PH, primary host plant; SH, secondary host plant. Other abbreviations are provided in Table S2.


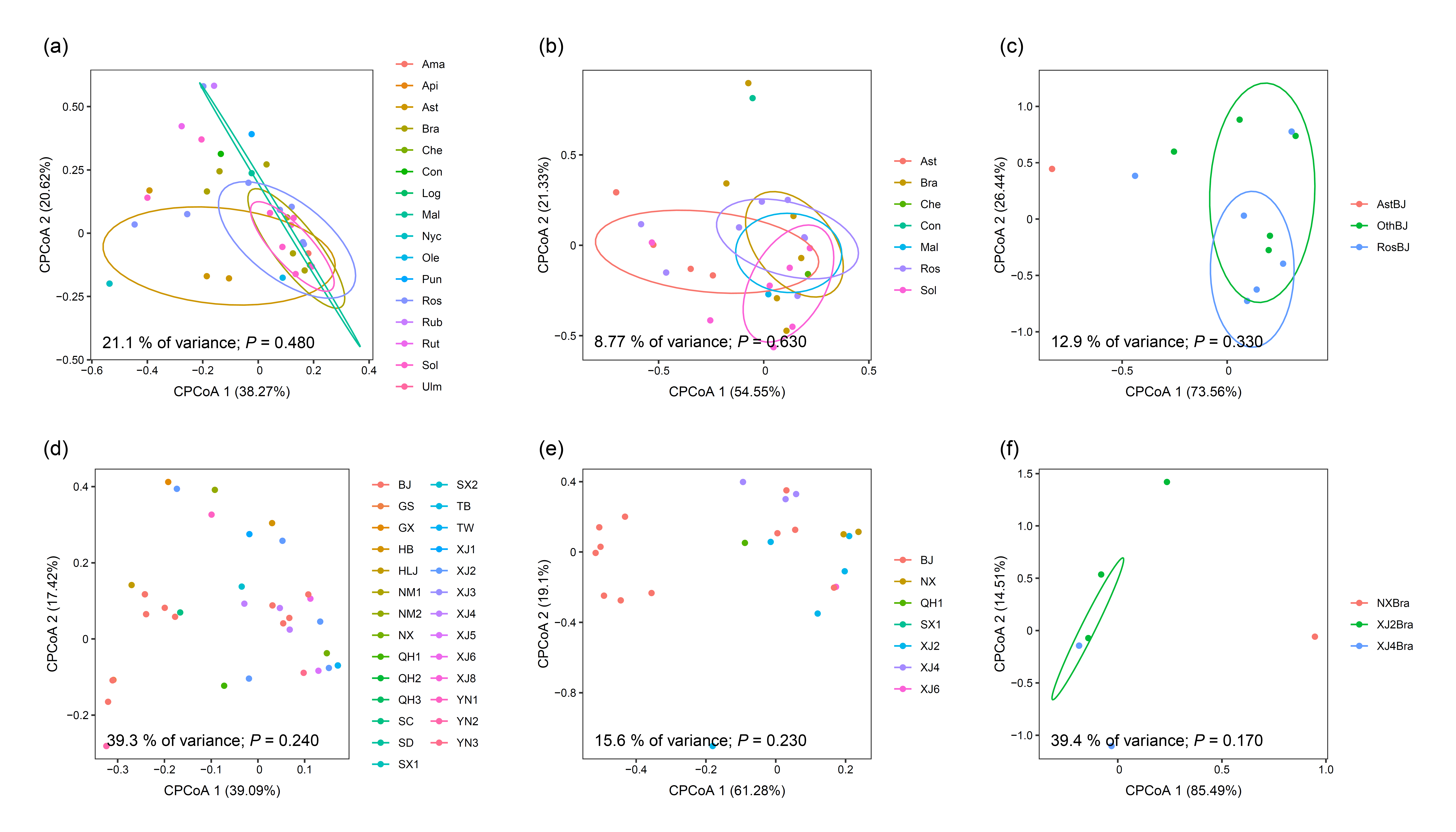


**Fig. S3** No significant variation observed in the secondary symbiont communities associated with *Myzus persicae* as revealed by constrained principal coordinate analyses (cPCoA) of Bray−Curtis distances at the OTU level. **a** All 16 host plant groups. **b** Seven host plant groups (n ≥ 3). **c** Three host plant groups from Beijing (n ≥ 3). **d** All 30 geographic groups. **e** Seven geographic groups (n ≥ 3). **f** Three geographic groups on Brassicaceae (n ≥ 3). Abbreviations are provided in Table S2.
